# Prevalence and risk factors for micronutrient deficiencies during pregnancy in Cayenne, French Guiana

**DOI:** 10.29219/fnr.v65.5268

**Published:** 2021-02-22

**Authors:** Amandine Duclau, Fanny Abad, Antoine Adenis, Nadia Sabbah, Malika Leneuve, Mathieu Nacher

**Affiliations:** 1 Centre d’Investigation Clinique, Centre Hospitalier Andrée Rosemon, Cayenne, French Guiana; 2 Service d’endocrinologie et diabétologie, Centre Hospitalier Andrée Rosemon, Cayenne, French Guiana; 3 Pôle Femme Enfant, Centre Hospitalier Andrée Rosemon, Cayenne, French Guiana; 4 Université de Guyane, Cayenne, French Guiana

**Keywords:** micronutrients, deficiencies, pregnancy, risks factors, social inequalities in health, French Guiana

## Abstract

**Background:**

Involved in physical and brain development, immunity and metabolism, micronutrients have profound health effects. The nutritional status of pregnant women is a major determinant of foetal health. French Guiana has a rapid population growth. Social inequalities, cultural practices and gastrointestinal nematode infections in French Guiana could affect the prevalence of these deficiencies. The main objective of the present study was to estimate the prevalence of micronutrient deficiency among pregnant women in French Guiana. The secondary objective was to identify socio-demographic, dietary, obstetrical and neonatal risk factors associated with deficiencies.

**Methods:**

Pregnant women over 22 weeks of pregnancy hospitalized for delivery at the Obstetrical Emergency Department of the Hospital Center in Cayenne from May 2018 to March 2019 were included. A socio-demographic and food questionnaire was administered. Medical data were collected from the medical records. Blood and urine samples were taken. The descriptive analysis used Student and chi-squared tests.

**Results:**

A total of 341 women were included. The majority were born in Haiti (39%) and French Guiana (34%). At least one micronutrient deficiency was observed in 81% of women. Precarious women had a significantly greater risk of micronutrient deficiency during pregnancy compared to those with both normal and complementary health insurance.

**Conclusions:**

Micronutrient deficiencies in pregnant women in French Guiana are a public health problem, a fact that was previously overlooked in the context of rising obesity. With over half the women overweight or obese, and 81% with at least 1 micronutrient deficiency, balanced nutrition should be a major focus.

## Popular scientific summary

- Micronutrient deficiencies are frequent in pregnant women in French Guiana, a French territory in South America. Micronutrient deficiencies are more frequent in precarious women. Obesity is a frequent problem but it is also associated with micronutrient deficiency. Micronutrient deficiencies have largely been overlooked in this outermost European region and should be corrected.

Micronutrients, also known as vitamins and minerals, are the essential components of a quality diet and have profound effects on health. Although they are only needed in very small quantities, micronutrients are the essential building blocks for stimulating human growth and metabolism. They are involved in physical and brain development, the functioning of the immune system and various metabolic processes (1, 2).

Micronutrient malnutrition is widespread in industrialised countries, and even more so in developing regions. It can affect all age groups, but young children and women of childbearing age tend to be most at risk (3). Micronutrient deficiencies can even occur in communities where the food supply is sufficient to meet the population’s energy needs. They are often referred to as ‘invisible hunger’: the body, despite a satisfactory caloric intake, is always hungry for good nutrition (4–6).

Pregnancy leads to increased metabolic needs, due to the physiological changes of the pregnant woman and the needs of the growing foetus. Micronutrient malnutrition is harmful for women and their fetus’ health (7). Not all of micronutrient malnutrition’s health consequences are clinically apparent. Even moderate deficiencies (detected by biochemical measurements) can seriously impair the functioning of the human body. Therefore, in addition to its direct effects on human health, micronutrient malnutrition has profound implications for economic development and productivity, particularly in terms of potentially high public health costs and losses in human capital formation.

According to World Health Organization (WHO), the most common micronutrient deficiencies are iron, vitamin A and iodine deficiencies. These are followed by zinc, folic acid (vitamin B9), vitamin B12 and other B-group vitamins, vitamin C, vitamin D, calcium, selenium and fluoride (4–7).

French Guiana’s 260,000 inhabitants constitute a heterogeneous group, including (but not limited to) Creoles, Maroons, metropolitans (French from mainland France), Amerindians, Hmong and immigrants from South America, the Caribbean and Asia (8).

A single road runs along the entire coastline from east to west. The rest of the territory is only accessible by canoe, plane or helicopter. Health coverage is thus heterogeneous and the health system suffers from a shortage of health professionals.

French Guiana has a very high average annual population growth (+3.1% between 2007 and 2030). French Guiana is the country with the highest fertility rate, along with Guatemala in Latin America. The high birth rate (3.5 vs. 2 in metropolitan France) is one of the drivers of this important demographic growth. In 2017, there were 4,321 births at the maternity ward in Cayenne Hospital.

The national perinatal survey estimated that in 2010, in the French overseas departments (Guadeloupe, French Guiana, Reunion Island), 12% of live singleton births occurred prematurely, whereas it was 5.5% in mainland France (9). In French Guiana, the preterm birth rate is stable around 13.5% of live births (10).

Additionally, in French Guiana, excess weight and obesity are a major public health problem that can mask the problem of malnutrition. Indeed, the lack of resources hampers the diversification of the diet of a large proportion of the population and leads to insufficient consumption of adequate quantities of fruits, vegetables or foods of animal origin containing micronutrients, and finally leading to deficiencies. Precariousness and social isolation are two of French Guiana’s major problems. They often led to renounced rights and care, underpinning health inequalities (11). In addition, digestive parasitoses or problems linked to the lack of safe drinking water and diarrheal diseases can lead to nutritional deficiencies by malabsorption.

In this context, where the potential causes of micronutrient deficiencies are frequent, the main objective of the present study was to estimate the prevalence of micronutrient deficiencies among women hospitalised for delivery. The secondary objectives were to analyse the socio-demographic and dietary risk factors associated with micronutrient deficiencies and to analyse obstetrical and neonatal consequences of micronutrient deficiencies.

## Methods

### Study design

The study was descriptive, cross-sectional and monocentric.

### Study site

Cayenne hospital was the center of inclusion because in 2017, it accounted for half of the births in French Guiana.

### Inclusion and exclusion criteria

Any woman who was pregnant (22 weeks and more), hospitalised for delivery in the Gynecological and Obstetrical Emergency Unit at Cayenne hospital could participate in the study after giving written, informed consent. The mode of delivery or urgent/non-urgent context did not influence the eligibility for inclusion. The criteria for non-inclusion were refusal to participate in the study; being under guardianship, and having already been enrolled in the study.

### Sample size

The required number of subjects was calculated so that the deficiency rate for each of the micronutrients studied could be estimated. A total number of 300 patients was considered sufficient for a 5% precision and a 95% confidence interval.

### Measurements

Micronutrient deficiencies were investigated by blood and urine sampling. The samples were analysed by the Biomnis laboratory in Paris. The results were then classified as deficiencies according to the biological thresholds defined by the WHO which are shown in Table 1.

**Table 1:** Definitions of micronutrient deficiencies

Micronutrient	Measurement level	Deficiency threshold*
**Cobalamin**	Blood	<150 pmol/L
**Folates**	Erythrocyte	<140 ng/mL
**Iodine**	Urine	<100 μg/L
**Iron**	Blood	Ferritin <15 μg/L Serum iron <5.8 μmol/L Transferrin iron saturation coefficient <16%
**Magnesium**	Blood	<0.66 mmol/L
**Retinol**	Blood	<0.70 μmol/L
**Zinc**	Blood	<700 μg/L

* Source World Health Organization

Concerning iron deficiency, ferritin, a recognised indicator for estimating iron deficiency, decreases during pregnancy. In order not to overestimate iron deficiency by using the hypoferritinemia criteria (may decrease during pregnancy), it was decided to assess iron reserves with different markers in order to define iron deficiency (Table 1).

### Socio-demographic information

Socio-demographic and dietary risk factors associated with micronutrient deficiencies were investigated using a structured questionnaire.

Socio-demographic data: age, place of birth, mother language, place of residence in the last 9 months, date of arrival in French Guiana, possession of a residence permit (for foreign women), health coverage, education level, marital status, professional status before pregnancy.

### Food data

Interviews were performed in French, Spanish, Creole, English and Portuguese. The food diversity score, inspired by the Food and Agriculture Organization’s reports (12) was assessed through a 24-h recall, and calculated by adding the number of unique food groups consumed during last 24 h. The 9 food groups were: cereals/roots, green vegetables, fruits and vegetables rich in vitamin A, other fruits and vegetables, legumes/lentils, offal, meat/fish, eggs, milk/dairy products. If an individual ate any quantity of any food group at least once per day, the group was taken into account. Each group accrued one point so the score range from 0 (no food) to 9 for consumption of all groups of food in the past 24 h. Therefore, the food diversity score was qualitative and did not entail a minimum intake for each food group. The quantitative conformity with the French Ministry of Health’s nutritional recommendations was evaluated (13). These recommendations suggest consuming certain quantities of servings per day, without specifying a clear general benchmark. Water consumption was investigated by asking them how many glasses/bottles of water they drank each day and from what origin the water came.

### Obstetrical and neonatal outcomes

Obstetrical and neonatal pathologies associated with micronutrient deficiencies were studied through patient interrogation and by analysing medical records.

### Medical data

Mother: weight before pregnancy and on the day of inclusion, height, gestational age, pregnancy follow-up, microcytic anaemia (haemoglobinemia <110 g/L, Mean corpuscular Volume >80 fl), primary hypothyroidism (increased Thyroid Stimulating Hormone), gynaecological-obstetric history, personal history of goiter or dysthyroidism, history of night blindness, personal history of anaemia, other chronic conditions, history of digestive disorder/parasitosis, history of malaria, drug treatment before and during pregnancy, micronutrient supplementation before and during pregnancy, tobacco use during pregnancy, alcohol use during pregnancy.

Newborn: gestational age at delivery, mode of delivery, weight, height, head circumference, appearance, pulse, grimace, activity and respiration (APGAR), and pathologies diagnosed on clinical examination before the 8th day of life.

### Study conduct

The inclusions were performed by the investigator from Monday to Friday between 7 AM and 5 PM according to the order of arrival of women at the gynaecological and obstetrical emergency unit of the Hospital in Cayenne.

On the first day of hospitalisation in the department, the investigator proposed the study to women who met the inclusion criteria. The investigator collected the patient’s data from the medical record and interviewed the patient to complete the nutritional questionnaire.

The required biological, blood and urine samples were collected during routine blood sampling upon entry and hospitalisation.

### Statistical analysis

Statistical analysis was performed with Stata13© (College Station, Texas, USA).

The descriptive analysis used Student’s t-test for quantitative data, Chi-squared test or Fischer’s test for qualitative data. A *p*-value of less than 0.05 was considered significant. The low percentage of missing data did not require any specific statistical intervention.

The variable ‘at least one micronutrient deficiency’ was cross tabulated with variables that reflected the socio-demographic characteristics in order to identify concrete potential intervention targets. Univariate and multivariate logistic regression was used to adjust for confounding.

### Ethical and regulatory aspects

The study received ethical approval (Agence Nationale de Sécurité du Médicament 2018-A00139-46, Comité de Protection des Personnes ile de France V, and the Commission Nationale Informatique et Libertés); all women received information about the study and gave consent for analysis and publication of the results and conclusions.

## Results

A total of 341 pregnant women admitted to the Obstetrical Emergency Department who gave birth at the hospital in Cayenne agreed to participate in the study between 29 May 2018 and 29 March 2019.

### Socio-demographic characteristics

The average age was 28.6 years (range 15–45 years). The majority of women were born in Haiti and French Guiana (39 and 34% respectively), 9% of them were born in Brazil, 6% in mainland France, 4% in Suriname and 4% in the Dominican Republic.

The mother language was Haitian Creole for 37% of the women, French for 31%, Portuguese for 11%, Nengue Tongo for 7%, Spanish for 4% of them.

The educational level of women participating in the study was ‘high school’ level for 35%, ‘middle school’ level for 31%, Twenty-one percent had completed higher education, 5% had vocational training and 8% of women had never attended school, or had interrupted their primary education.

Over a third of women were single (39%), 43% lived as a couple, 16% were married, and 2% were divorced.

The median length of stay in French Guiana at inclusion was 2.7 years (range 1 month–41 years) and 90% of the women participating in the study lived in French Guiana throughout their pregnancy.

Among foreign women, 68% did not have a residence permit.

Concerning health insurance coverage, 160 women (47%) benefited from the universal medical coverage, 68 (20%) from regular health insurance and a complementary health insurance, 66 (19%) from the State Medical Aid for foreigners, 21 (6%) from regular health insurance without complementary insurance, and 26 (8%) did not benefit from any coverage.

The majority of women (59%) were housewives, 26% had a permanent job, 4% were temporary workers, 7% were in training, and 5% were looking for work.

### Nutritional characteristics

The majority of women drank tap water (65%); 30% drank bottled water; the remaining 5% drank water from a well, rain or creek. Water intake was insufficient, under 1.5 L of water per day for 22% of women.

The 9-category food diversity score showed 52% of the women had a score of 4 or less.

The national nutrition and health program includes four items: 9% of women responded to the four items. 66% of women responded to two items or less. The most consumed foods where starchy foods (96%) and the least consumed were fruits and vegetables (42 and 33%, respectively) with 83.5% having less than the recommended five servings per day.

Thirteen percent of women experienced eating disorders during pregnancy, 11% consumed ‘pemba’, which consists of aluminum-rich clay. Among autochthonous populations living in the interior, Maroon women were more likely to declare that they consumed clay than Amerindian women; coal or soil (37% vs. 0%, *P* = 0.04, respectively).

The body mass index before pregnancy averaged 26.7. At the beginning of pregnancy, half of women had an abnormal body mass index (BMI): one in four women was overweight and one in four was obese.

The median weight gain during pregnancy was 10 kg. A quarter of women gained 15 kg or more during pregnancy, with a maximum of 37 kg.

### Obstetrical characteristics

The gestational age at inclusion ranged between 22 and 42 weeks of pregnancy. The mean gestational age was 36.5 weeks of pregnancy (±4.2). Fifteen percent of women gave birth prematurely, and 4.5% extremely prematurely. Fifteen percent of the newborns in the study had a birth defect, or required intensive care.

The first obstetrical consultation took place before the 14th week of amenorrhea for 75% of women. The average number of previous pregnancies was three. Among the 10% of primigravida women, 18% received prenatal consultations and 27% birth preparation sessions. Among multiparous women, 81% of them had breastfed their previous child.

Twenty-three percent of the women participating in the study had a history of voluntary termination of pregnancy. Almost one woman in four (24%) had a history of miscarriage.

Night blindness during pregnancy was reported by 11% of the women participating in the study.

### Biological characteristics

Microcytic anaemia was reported in 21% of women. Primary hypothyroidism was observed in 6.4% of women. For urinary iodine, the median observed in our study sample is 104 μg/L.

The details of micronutrient deficiencies are shown in Table 2.

**Table 2:** Number and proportion of micronutrient deficiencies

Micronutrient	Micronutrient deficient *n/N* (%)
Cobalamin	129/333 (39)
Folates	5/336 (1.5)
Iodine	115/243 (47)
Iron	37/335 (7)
Magnesium	37/336 (11)
Retinol	109/309 (35)
Zinc	5/334 (28)

At least one micronutrient deficiency was objectively detected in 81% of the women participating in the study; 46% of women had at least two deficiencies; and 18% had at least three.

No treatments known to induce deficiency were reported among the women in the study.

Table 3 showed that place of birth, mother language, health insurance, maternal education level were significantly associated with micronutrient deficiency. More specifically, the results in Table 3 showed that socially deprived women of foreign origin, having limited education were more at risk of micronutrient deficiency. The APGAR at 1 min score was lower among such women with at least one micronutrient deficiency, but this was not statistically significant (Table 3).

**Table 3:** Variables associated with at least one micronutrient deficiency

Characteristics	Univariate analysis		
	Prevalence ratio	IC 95%	*P*
Health insurance			0.011
Social and supplementary health insurance	1		
State Medical Aid	1.35	1.12; 1.63	0.002
Universal Medical Coverage	1.31	1.10; 1.57	0.003
None	1.22	0.95; 1.57	0.123
Social insurance	1.01	0.71; 1.43	0.967
APGAR 10			
	0.98	0.97; 0.99	0.011
Place of birth			0.026
French Guiana	1		
Brazil	0.94	0.74; 1.19	0.608
Guyana	0.98	0.55; 1.74	0.945
Haiti	1.17	1.04; 1.31	0.009
Metropolitan France	0.76	0.51; 1.13	0.169
Dominican Republic	1.21	1.00; 1.45	0.049
Surinam	1.13	0.91; 1.42	0.274
Educational status			0.001
High school	1		
Vocational school certificate	0.82	0.60; 1.12	0.208
Bachelor degree	0.85	0.71; 1.01	0.061
Non-response	0.58	0.14; 2.32	0.440
Secondary school	0.99	0.89; 1.10	0.898
Doctorate	0.01	0.01; 0.01	0.001
Master degree	0.62	0.37; 1.04	0.068
Never attended school	1.16	1.08; 1.24	0.001
Primary school	0.90	0.80; 0.93	0.421
Mother language			0.002
French	1		
Amerindian	0.79	0.41; 1.52	0.485
English	0.92	0.52; 1.65	0.790
French Guiana creole	1.13	0.84; 1.54	0.415
Haitian creole	1.24	1.09; 1.42	0.001
Spanish	1.29	1.07; 1.55	0.009
Portuguese	1.00	0.79; 1.27	0.990
Nengue Tongo	1.33	1.15; 1.54	0.001

The socio-demographic variables were re-coded in simpler categories. Table 4 shows that after adjustment with logistic regression models, only precariousness remained associated with the presence of at least one micronutrient deficiency.

**Table 4:** Multivariate analysis of socioeconomic variables associated with at least 1 micronutrient deficiency

Characteristics	Deficiency *N* (%)	No deficiency *N* (%)	Crude Odds Ratio (95%CI)	Adjusted Odds Ratio (95%CI)	*P*
**Precariousness**					
**Normal health insurance**	59 (66.3)	30 (33.7)	1		
**No health insurance/welfare (CMU/AME)**	216 (86.7)	33 (13.3)	3.3 (1.9–5.9)	2.7 (1.3–5.8)	0.009
**Place of birth**					
**French territory**	102 (37.2)	35 (55.6)	1		
**Foreign country**	172 (62.8)	28 (44.4)	2.1 (1.2–3.7)	1.1 (0.5–2.7)	0.8
**Educational status**					
**None/primary**	24 (8.8)	4 (6.4)	2.7 (0.85–8.9)	1.27 (0.3–4.6)	0.7
**Secondary**	202 (73.7)	36 (58)	2.6 (1.4–4.7)	1.67 (0.8–3.3)	0.14
**University**	48 (17.5)	22 (35.5)	1	1	
**Mother language**					
**French**	74 (27)	29 (46.8)	1	1	
**Other**	200 (73)	33 (53.2)	2.4 (1.3–4.2)	1.05 (0.4–2.7)	0.9

*Pearson’s goodness of fit test, *P* = 0.7.

## Discussion

Our study reveals that micronutrient deficiencies are a real public health problem for pregnant women in French Guiana. Indeed, there was at least one confirmed micronutrient deficiency in 81% of the women participating in the study.

The measurements in our study in relation to the literature indicate that the public health problem in French Guiana’s population of pregnant women concerns iron deficiency and its complication: anaemia, vitamin A deficiency and iodine deficiency (4). The lack of consensus for the other micronutrients analysed does not allow us to state that the observed deficiencies constitute a public health problem. Nevertheless, our study also shows the substantial prevalence of vitamin B12 (39%), zinc (28%) and magnesium (37%) deficiency (4).

Our study found anaemia in 41% of women. The decreased hemoglobin because of physiological haemo-dilution during pregnancy cannot be considered as a justification for anaemia below the threshold of 11 g/dL during the 3rd trimester of pregnancy. The consumption of ‘pemba’ clay is known to promote anaemia and 11% of the women in our sample reported using it during their pregnancy (14). However, this consumption was discontinued beyond the first trimester of pregnancy.

There was a 35% prevalence of vitamin A deficiency in our population; additionally, night blindness was reported by 11% of the study participants. As this prevalence is higher than 5%, it is an additional criterion defining a public health problem (4). The prevalence of Vitamin A deficiency and night blindness seemed substantially greater than what is reported in Latin America, and is comparable to that reported in Africa and South Asia (15).

In our study sample the median urinary iodine concentration was 104 μg/L and primary hypothyroidism was documented in 6.4% of participants. This is below the median urinary iodine concentration of 150 μg/L in pregnant women among the general population; thus defining it as a public health problem (16).

Magnesium deficiency is difficult to define and there is no consensus to date on a reliable biological indicator (16). However, in the SU.VI.MAX study conducted in France under the supervision of INSERM, the analysis of the magnesium dietary intakes of about 5,500 people revealed that about 3/4 of them had insufficient intakes.

By contrast, the prevalence of folic acid deficiency was found to be low (1.5%). This suggests good compliance with the recommendation of systematic supplementation (5 mg/day) during the first trimester of pregnancy. This suggests that for other micronutrients, if we set clear nutritional goals for French Guiana, recommendations could reach their target irrespective of the women’s social vulnerabilities.

The women most at risk of developing these deficiencies were women born in Haiti and the Dominican Republic, who often lived in precarious conditions. Given the context of intense immigration from Haiti to French Guiana, facilitating access to care soon after these women arrive should be a priority. Among autochthonous populations living in the interior, the prevalence of micronutrient deficiencies seemed more prevalent in Maroon women than in Amerindian women. These women were more likely to declare that they consumed clay, coal or soil which could partly explain the observed difference along with cultural dietary differences.

Over half of the women in the study were overweight (27%) or obese (27%) at the beginning of their pregnancy. This excess weight masks a diet that was found to be low in nutritional intake. Indeed, 52% of the women interviewed had a food diversity score of 4 or less. The consumption of 66% of the women participating in the study responded to only 2 nutritional categories out of 4, or less; which is not enough according to the recommendations of the French National Nutrition Health Programme (9, 17). Although gestational diabetes is frequent among pregnant women in Cayenne Hospital (10.3%), the hypothesis that reduced intake would lead to deficiencies seems implausible given the considerable difference in magnitude of the problem.

These results suggest that it is important and urgent to set up nutritional support for all women in French Guiana. In addition, nutritional education in school may also promote a varied and balanced diet, in order to prevent obesity and morbidity in adulthood, at least for those born in French Guiana. However, the barrier to a balanced nutrition is not necessarily related to knowledge gaps, and is often attributed simply to the high costs of a varied diet and perhaps a lack of time for preparation.

In French Guiana, in 2015, 12.8% of newborns had a low birth weight versus that of 8.2% in metropolitan France. The national perinatal survey in 2010 estimated that in French overseas departments (Guadeloupe, French Guiana, Reunion Island), 12% of live singleton births occurred prematurely versus 5.5% in mainland France. The prematurity rate in French Guiana is stable at 13.5% of live births (18).

This emphasizes the singularity of health problems in French Guiana relative to in mainland France. Prevention and medical care for women and children must thus be designed and adapted to the particularities of this region.

The study sampled women arriving in the Obstetrical Emergency Unit at Cayenne Hospital. Although this sample does not represent all pregnant women in French Guiana, the majority of the population uses the hospital in Cayenne to deliver their babies. Our sample size corresponded to the calculated optimal theoretical sample size. In addition <1% of eligible women refused to participate in the study and none were lost to follow-up. Given these elements, we consider that the representativeness of the study was good.

However, there were possible study limitations: laboratory constraints, restrained inclusions during weekday working hours. Our sample represented 10% of the total number of births over the 10-month inclusion period. It is likely that women consulting during the day may present pregnancies at higher risk of delivery without the patient actually being in labor.

The inclusions were also found to be dependent on the on-call medical team. Some women already in labour presenting themselves in the unit for an imminent delivery were not requested to participate in the study due to lack of time.

Thus, the proportion of pathological pregnancies was probably slightly overestimated. In addition, the sample probably underestimated the proportion of underage women. Although they were willing to participate in the study, some could not be included in the study because of the lack of representation of a legal guardian. These underage women came most often from isolated villages.

The food data collection method used was the 24-h recall method. This is the most common and recommended method to limit memory bias (12, 19, 20). Three different investigators collected the data. The anticipation of a measurement bias that could be induced by the investigator’s subjectivity was limited by the introduction of a procedure for conducting the questionnaire and collecting medical data. In addition, the investigators were trained in pairs for the necessary time. In addition, our study included a reporting bias due to the difficulty of communicating with women who do not speak French at all. However, this only affected a small percentage of our sample.

To our knowledge there have not been studies in Latin America comprehensively documenting these deficiencies, and overall single micronutrient data is scarce (21).

Although, it is difficult to generalize from sketchy data collected at different times in different populations, with different methods, it seemed that in Latin America the prevalence of some micronutrient deficiencies is lower than in the rest of the tropical world (15). It also seemed that despite the widespread poverty in French Guiana, micronutrient intake was generally greater than in African or Asian tropical countries, leading one to expect that in a territory with the highest Gross Domestic Product per capita it would be even greater (22). But the data presented here show that there is indeed a micronutrient deficiency problem, and that it is much more prevalent than expected and hence, requires to be acted upon.

## Conclusion

Although the fight against obesity should still be of major public health importance, we here show that the nutritional problems in French Guiana are complex with excesses and deficiencies; a fact that was overlooked until now and is of great operational importance. Indeed, folate deficiency was rare which suggests that, when a micronutrient is specifically targeted, supplementation efforts may have an impact in our population. Thus targeting other micronutrients should also be possible in our population. The present results should surely raise awareness and guide specific efforts to rapidly compensate these deficiencies. A varied and balanced diet remains the most effective solution to prevent these deficiencies from occurring (23). However, poor populations often cannot afford, or do not know how to diversify their nutritional intake. This segment of the population is particularly at risk for both micronutrient deficiencies and obesity, and should thus be the target of nutritional interventions.
